# Influence of hospitalization on prescribing safety across the continuum of care: an exploratory study

**DOI:** 10.1186/s12913-015-0844-x

**Published:** 2015-05-13

**Authors:** Wilko von Klüchtzner, Daniel Grandt

**Affiliations:** University of Duisburg-Essen, Essen, Germany; Hospital of Saarbrücken, Saarbrücken, Germany

**Keywords:** Medication, Prescribing, Safety, Hospital, Admission, Discharge

## Abstract

**Background:**

Transitions between different levels of healthcare, such as hospital admission and discharge, pose a considerable threat to the quality and continuity of drug therapy. This study aims to further explore the current role of hospitalization in prescribing error exposure and medication-related communication as patients are transferred from and back to ambulatory care.

**Methods:**

Assisted by electronic decision support, pre-admission and discharge medication regimens of 187 adult patients in a German university hospital were comparatively screened for clinically relevant categories of potentially inadequate prescribing. Binary logistic regression analyses were conducted to identify risk factors predisposing individuals to prescribing errors as a result of hospitalization. Additionally, it was established to what extent medication changes and potentially inappropriate prescribing decisions originating from inpatient treatment were communicated in discharge letters.

**Results:**

94.7% of the patients are subjected to differences between pre-admission and discharge prescriptions occurring at a rate of 461 per 100 hospitalizations. However, these modifications in drug therapy do not have a significant effect on the total number of potential prescribing errors per patient (p = 0.135) even though a large potential for improvement exists throughout the care continuum. For instance, almost a quarter of study participants with impaired kidney function lacks appropriate dose adjustment for one or more drugs before onset and at the end of inpatient treatment alike (22.5% [95% CI: 13.5%-34.0%] vs. 22.8% [95% CI: 14.1%-33.6%]). Overall, the probability of error exposure following hospitalization rises with an increasing number of prescribed drugs per patient, while individuals treated on surgical wards are four times more likely to be discharged with a prescribing-related safety hazard than their counterparts from medical departments (OR: 4.069 [95% CI: 1.126-14.703]; p = 0.032). In the study population’s discharge summaries only 14.8% of medication changes and none of the potentially inappropriate prescribing decisions made during inpatient care are addressed, despite the latter occurring at a rate of 91 per 100 hospitalizations.

**Conclusions:**

There is urgent need for standardized and evidence-based measures contributing to patient safety across sectorial interfaces of drug therapy. Our findings provide useful orientation for the targeted and rational design of such improvement strategies.

## Background

Transitions between different levels of healthcare, such as hospital admission and discharge, pose a considerable threat to the quality and continuity of drug therapy. This is primarily due to poor management and transfer of information both between health professionals and in relation to their patients [1,2]. These shortcomings gain significance as increasingly complex medication regimens tend to be under fragmented control of various specialized caregivers with limited resources for coordination and documentation of treatment plans. Moreover, differing drug formularies as well as unsteady and vulnerable patient conditions are typical phenomena at care interfaces that further add to the challenge. In line with this, a prospective, observational cohort study from Denmark has found medication data retrieved from the patient, the general practitioner and the hospital to show complete consistency for only 8% (95% CI: 3%-17%) of consecutive medical cases throughout the continuum of care [3].

Despite the aforementioned problems, a hospital stay allows for intensive medical assessment and monitoring, thus providing an opportunity to adjust and coordinate all medication-related aspects for the benefit of cross-sectorial patient safety. With a view to determining to what extent current hospital practice lives up to this potential, the present study explores the role of hospitalization in prescribing error exposure and medication-related communication as patients transition from and back to ambulatory care. This may consequently assist in informing the rational development of targeted improvement strategies.

## Methods

This exploratory study took place at Essen University Hospital, a large urban academic medical centre in Germany, after approval had been granted by the institution’s ethics review committee^a^. In accordance with preliminary power calculations (Table 1) we recruited 200 patients from a sample of medical and orthopaedic/surgical wards between mid-June and mid-October 2011. Due to time constraints it was not possible to visit all of the wards on a daily basis. However, for each visited ward recruitment was conducted consecutively according to the patients’ order of admission on that given day, as long as exigencies of clinical routine care would not stand in the way. Considered for inclusion were patients aged 18 years or older, being treated with at least one previously prescribed drug at the time of admission and giving informed consent to participate in the study. Transfers from and/or to other institutional health care facilities were excluded. Only one hospitalization was taken into account for each individual study participant, irrespective of any subsequent readmissions.

**Table 1:** **Calculation of study power**

**Difference of the means**	**Standard deviation**	**Power**
1.0	3.0	99%
	3.5	95%
	4.0	89%
	4.5	81%
1.1	3.0	>99%
	3.5	98%
	4.0	94%
	4.5	87%
1.2	3.0	>99%
	3.5	99%
	4.0	97%
	4.5	92%
1.3	3.0	>99%
	3.5	>99%
	4.0	99%
	4.5	96%

The primary hypothesis for power calculation implied that the mean number of prescribed drugs per patient would increase during hospital stay. Relying on preliminary investigations into the distribution pattern of prescriptions at admission to Essen University Hospital (mean number of prescriptions ± standard deviation: 7 ± 3) and assuming correlations of r = 0.2 to r = 0.7 between the mean prescription numbers at admission and discharge, the standard deviation of the difference between them was deduced to take on values between 3.0 and 4.5. The difference between the means itself was assumed to be at least 1. Based on these assumptions the primary hypothesis was tested in varying scenarios using matched-pair signed-rank test with a significance level of 5%. With a set sample size of n = 180 a study power of more than 80% was achieved in each tested scenario. To account for a drop-out rate of up to 10% the needed number of participants was determined to be 200.

### Data collection

Prospective data collection comprised systematic and integrative collation of comprehensive medication profiles across the transitions to and from hospital including corresponding patient and morbidity characteristics. Following a standardized protocol^b^, we initially obtained best-possible home medication histories by interviewing patients, their families and/or carers. Medication plans and/or supplies carried by the interviewees were also taken into account. In addition, we sought any existing referral letters from community care physicians as well as routine admission notes and other relevant medical records from present or past hospitalizations. Hospital laboratory test results required for prescribing safety appraisal (e.g. MDRD estimates of glomerular filtration rates [4]) were accessed electronically. On a regular basis, we contacted health professionals involved in the ambulatory care of study participants in order to resolve perceived gaps or inconsistencies in collected information. Finally, we retrieved electronic copies of the discharge summaries including recommendations on how to resume outpatient drug therapy.

### Data assessment

Assisted by a commercial clinical decision support software^c^, a clinical pharmacist comparatively reviewed individual pre-admission and discharge medication regimens (including all systemically active drugs for acute or chronic, regular or *pro re nata* use under medical control). This evaluation targeted the following clinically relevant categories of potentially inadequate prescribing that had been pre-defined drawing from an evidence-based range of explicit and implicit assessment criteria [5].

- Inappropriate dosing (e.g. with respect to impaired kidney function)
- Adverse or redundant combination of drugs
- Contraindicated drug choice
- Unjustified omission of an indicated drug
- Medication without indication
- Inappropriate drug choice for the therapeutic goal
- Medication for a patient aged 65 or older which is acknowledged as potentially inappropriate in this age group [6]
- Prescription of inappropriate tablet fractions

Each drug prescribed to a given patient could be possibly associated with more than one kind of inadequacy simultaneously. In case of potential uncertainties of classification consensus between the study authors was sought.

Moreover, for each study participant the number of discrepancies between pre-admission and discharge medication (i.e. temporary or permanent introduction or withdrawal of a drug, alteration of dose and/or frequency including switching between regular and *pro re nata* application schemes, therapeutic drug substitutions) was determined. It was established to which extent such medication changes as well as potentially inappropriate prescribing decisions (see categorization above) originating from hospital care were communicated in the discharge letter.

### Statistical analysis

95% confidence intervals were calculated according to Clopper-Pearson assuming binomial distributions. Gender allocation of the study sample was tested for agreement between observed and hypothesized binary probability. Depending on the scale of measure, the following tests were used for longitudinal comparisons of patient characteristics and prescribing error exposure at admission versus discharge: McNemar’s test for dichotomous variables, Marginal Homogeneity test for multinomial criteria and Wilcoxon matched-pair signed-rank test for metric parameters. To identify risk factors for potentially inadequate prescribing as a result of hospitalization, binary logistic regression analyses were conducted using a stepwise approach. In all adopted statistical procedures a significance level of α = 0.05 was applied.

## Results

**Figure 1:**
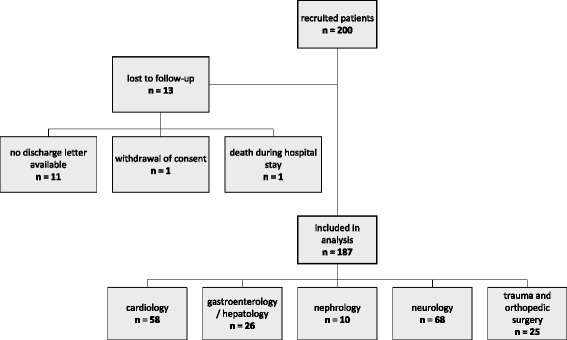
Results of patient recruitment. Reasons for patient exclusion and distribution of included patients to the different medical and surgical departments.

Out of 200 recruited patients, 187 (93.5%) are included in the evaluation while the remaining 13 (6.5%) are lost to follow-up (Figure 1). The majority of exclusions affecting on average 1 in 18 (5.6%) discharged study participants is due to missing discharge letters.

### Patient characteristics

The study population comprises both genders in comparable proportions (47.6% [95% CI: 40.3%-55.0%] women vs. 52.4% [95% CI: 45.0%-59.7%] men; p = 0.559). Both at admission and discharge two out of five patients (38.0% [95% CI: 31.0%-45.3%] vs. 42.2% [95% CI: 35.1%-49.7%]; p = 0.077) suffer from impaired kidney function (GFR < 60 ml/min/1.73 m^2^). More than one third (36.9%) of the participants is 65 years of age or older. Throughout the hospital stay a statistically significant increase of patients with more than 5 prescribed drugs is observed (52.9% [95% CI: 45.5%-60.3%] vs. 59.9% [95% CI: 52.5%-67.0%]; p = 0.043).

### Influence of hospitalization on prescribing safety across the care continuum

Only 1 out of 19 (5.3%) patients receives the same medication regimen both before entering and upon leaving the hospital. The predominant rest of the patients is exposed to differences between pre-admission and discharge prescriptions occurring at a rate of 461 per 100 hospitalizations. Nearly 9 out of 10 (89.3%) affected patients encounter multiple (up to 14) medication changes.

However, these extensive modifications in drug therapy apparently do not have an effect on overall prescribing safety (Table 2). Before admission and at discharge alike, nearly two thirds of study patients suffer from potentially inadequate prescribing, respectively. Similarly, the total number of potential prescribing errors per patient does not change significantly, either.

**Table 2:** **Overall burden of potentially inadequate prescribing at hospital admission and discharge**

**Parameter**	**Admission**	**Discharge**	**p-value**
Median (IQR) of potential prescribing errors per patient	1 (0–2)	1 (0–3)	0.135
Number of patients with prescribing error exposure	118	122	0.651
single error	44	44	0.454
multiple errors	74	78	

IQR: interquartile range.

**Figure 2:**
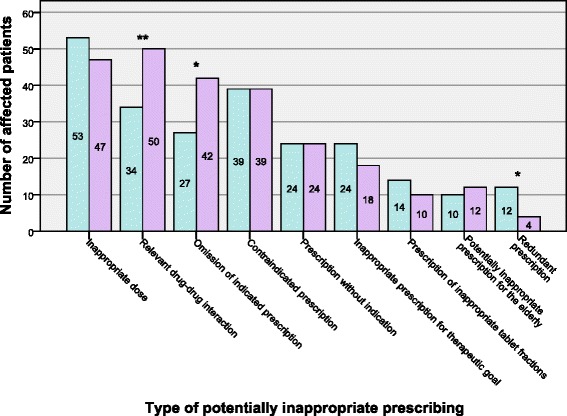
Number of patients affected by specific types of potentially inadequate prescribing at admission (blue bars) versus discharge (purple bars). *: p < 0.05; **: p < 0.01.

**Figure 3:**
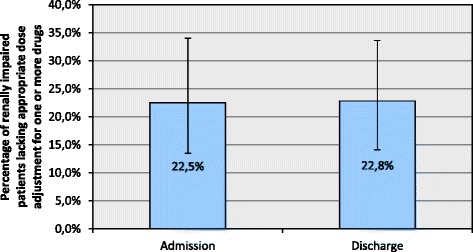
Percentage of renally impaired patients lacking appropriate dose adjustment at admission versus discharge. Error bars: 95% confidence intervals.

However, an in-depth analysis of the error pattern that patients are exposed to reveals significant alterations associated with hospitalization (Figure 2). Clinically relevant drug-drug interactions as well as omissions of indicated medication affect more patients at discharge than at admission. In contrast, merely the relatively small frequency of patients with redundant prescriptions is further reduced during inpatient treatment. Meanwhile, patient exposure to other, more prevalent types of potentially inadequate prescribing such as dosing errors are not altered significantly between admission and discharge. For instance, at both points in time almost a quarter of patients with impaired kidney function lacks appropriate dose adjustment for one or more drugs (Figure 3).

Two independent risk factors that predispose to the occurrence of potentially inadequate prescribing as an outcome of hospital care are shown in Table 3. The probability of error exposure rises with an increasing number of prescribed drugs per patient, and individuals treated on a surgical ward are four times more likely to be discharged with a prescribing-related safety hazard than their counterparts from medical departments.

**Table 3:** **Binary logistic regression analysis**

**Variable**	**Univariate analysis**		**Multivariate analysis**	
	**Odds ratio (95% CI)**	**p-value**	**Odds ratio (95% CI)**	**p-value**
Gender				
male	1.959 (1.064-3.608)	0.031	2.115 (0.970-4.611)	0.060
female (reference)	−	−	−	−
Age (years) at discharge	1.043 (1.022-1.064)	<0.001	1.017 (0.988-1.047)	0.258
GFR (ml/min/1.73 m^2^) at discharge	0.973 (0.958-0.988)	0.001	1.000 (0.978-1.023)	0.988
Prescribed drugs per patient at discharge	1.551 (1.355-1.777)	< 0.001	1.524 (1.297-1.790)	< 0.001
Length of stay (days)	1.044 (0.988-1.104)	0.122	0.980 (0.902-1.064)	0.631
Department				
surgical	2.353 (0.840-6.594)	0.104	4.069 (1.126-14.703)	0.032
medical (reference)	−	−	−	−

(Incremental) impact of selected patient and treatment factors on the probability of exposure to potentially inadequate prescribing associated with hospitalization (CI: confidence interval).

Finally, medication-related communication in the discharge letters of the study population has been found to be highly incomplete in that only 1 out of 7 (14.8%) alterations to the pre-admission medication regimen and none of the potentially inappropriate prescribing decisions made during inpatient treatment are pointed out or justified even though the latter occur at a rate of 91 per 100 hospitalizations.

## Discussion

In line with findings of an investigation conducted in Northern Ireland [7], the present study reveals no statistically significant improvement in overall prescribing safety as a result of routine inpatient treatment even though a large potential for error reduction is apparent throughout the care continuum. While the total extent of potentially inadequate prescribing is shown to remain constant between admission and discharge, our analyses convey marked alterations in the pattern of patient-level error exposure, thereby expanding the evidence published so far. For example, the prevalence of patients whose medication regimen lacks at least one indicated drug increases significantly by eight per cent until the end of the hospital stay. In nearly one out of four discharge prescriptions such an unjustified omission has been identified. Similarly, in their assessment of discharge summaries from an internal medicine department within a Swiss teaching hospital Perren and colleagues have found over a quarter of patients to be affected by this type of prescribing error [8].

Our findings further reveal that surgical patients are four times more likely to be discharged with potentially inadequate prescriptions than their counterparts from medical wards. This independent risk factor might reflect that surgical hospitalizations are primarily focused on acute interventions as opposed to the on-going pharmacotherapeutic management of comorbidities. This can be particularly problematic as surgery and follow-up care themselves regularly require (temporary) modification and/or expansion of usual home medication with additional implications for prescribing safety. In this regard, Boeker and colleagues note that the relative risk for post-operative complications is elevated almost by factor three (2.7; 95% CI: 1.8-4.0) in those patients taking co-medication beyond the context of their surgical treatment [9].

Our findings also confirm that medication-related information included in discharge letters of study participants is insufficient to ensure safety and continuity of drug therapy across different care settings. Identified shortcomings in communication concern both medication changes [10,11] and, as this study has additionally revealed, potentially inadequate prescribing decisions. Importantly, this impairs the ability of ambulatory care professionals to provide for an appropriate continuation of post-hospital drug treatment and latently facilitates occurrence and perpetuation of secondary prescribing errors [12]. Consequentially, these inadequacies are in stark contrast to community care providers’ informational demands as reported in the literature. For example, almost nine out of ten general practitioners anonymously responding to a survey in the catchment area of a Dutch urban teaching hospital wish to know whether and why inpatient medication changes have been introduced, and also appreciate clinical advice pertaining to prescribing safety [13].

Our study has a number of limitations. Representativeness and generalizability of our findings are limited by the study setting. Patients treated in a university hospital like ours tend to have more advanced healthcare needs than inpatients in general, which will likely affect the complexity of prescribing patterns and by extension may result in increased manifestation of related inadequacies [14]. There are also limiting implications of our study being monocentric, which may have been mitigated to only some extent by the broad range and diversity of patient cases recruited from various medical and surgical disciplines regardless of any morbidity specific restrictions. Meanwhile, patient recruitment has been compromised by the time and effort required for collecting scientifically sound data in an environment of clinical routine practice. Thus, it has not been possible to approach each and every admitted patient for inclusion in the study. We believe, however, that this has not led to a systematic distortion of our findings because on each occasion any given study ward was visited patients were recruited consecutively in their order of admission. Finally, our approach to pre-define targeted categories of inadequate prescribing, while lending itself to computational operationalization of measurement and enhancing objectivity and reproducibility of obtained results, will not be able to account for each and every form of insufficient prescribing safety despite the context sensitivity of the assessment criteria applied. However, this limitation may be deemed acceptable in the light of observations made by a systematic review of the medication error literature challenging the assumption of an automatically given association between the number of error types considered on the one hand and the overall error prevalence detected on the other [15].

## Conclusions

Our findings highlight the urgent requirement to develop and implement standardized and evidence-based measures contributing to patient safety across sectorial interfaces of drug therapy. At admission, a best-possible medication history matching drugs and corresponding diagnoses should routinely be obtained and made centrally available in order to review quality and coherence of the underlying prescribing decisions with regard to the current healthcare needs of the individual patient. This may subsequently inform inter-professional coordination of further inpatient medication management, paying particular attention to poly-medicated and/or surgically treated patients as a possible means to rationalize improvement efforts. At discharge, detailed recommendations for continued drug treatment including insights on medication changes and/or prescribing-related safety hazards possibly requiring special follow-up monitoring ought to be transmitted to subsequent health care providers through appropriate means of communication. Given restrictions in time and staff resources, the intelligent use of IT solutions holds promise to support and control implementation of the aforementioned approaches for the benefit of trans-sectorial prescribing safety.

## Endnotes

^a^Committee of the Medical Faculty of the University of Duisburg-Essen

^b^The standardized protocol was based on a structured data collection form detailing demographic, clinical and medication-related information required for each patient from pre-admission through to discharge. Conceptual development of this tool relied on a comprehensive review of the pertinent literature and of outputs from publicly advocated initiatives pursuing seamless medication safety (e.g. resources from the *Safer Healthcare Now!* campaign, the *Institute for Healthcare Improvement*, the *Agency for Healthcare Research and Quality*, etc.). Prior to implementation in this study, practicability and functionality of the resulting procedure were tested and refined following a stepwise approach in a sample of 100 patients.

^c^The tool used is a component of the software product RpDoc® (version 2.4.2) and as such provides informational support for medication prescribing and review with a particular focus on dosing, interactions and redundancies, contraindications and galenical issues (e.g. breakability of tablets). The user is automatically alerted to potential prescribing errors of clinical relevance and can access evidence-based explanations and recommendations for optimized use of each drug. All information provided is tailored to the specific therapeutic context by accounting for individual patient and morbidity related characteristics (e.g. age, sex, body weight, lab results, indications). The mentioned functionalities are based on regularly updated databases comprising contents extracted from the summaries of product characteristics, publications from regulatory and other healthcare related authorities, guidelines from medical and pharmaceutical associations and societies or their respective drug commissions, etc.
